# Managing comorbidities in chronic kidney disease reduces utilization and costs

**DOI:** 10.1186/s12913-023-10424-8

**Published:** 2023-12-15

**Authors:** Yong Li, Kanchan Barve, Meghan Cockrell, Amal Agarwal, Adrianne Casebeer, Suzanne W. Dixon, Insiya Poonawalla

**Affiliations:** 1 Humana Healthcare Research, Humana Inc, 500 W Main Street, Louisville, KY 40202 USA; 2 Humana Inc, 500 W Main Street, Louisville, KY 40202 USA; 3 Affiliated with Humana at the Time of the Work, Current Affiliation Monogram Health, Brentwood, TN USA

**Keywords:** Chronic kidney disease, Healthcare resource utilization, Diabetes care, Hypertension, Care coordination, Managed care

## Abstract

**Background:**

Effective management of comorbid diabetes and hypertension in patients with chronic kidney disease (CKD) is important for optimal outcomes. However, little is known about this relationship from a health plan perspective. The objective of this study was to evaluate the association of effective management of comorbid diabetes and/or hypertension with healthcare resource utilization (HCRU) in patients with chronic kidney disease (CKD).

**Methods:**

This retrospective cohort study used the Humana Research Database to identify patients with CKD Stage ≥ 3a in 2017. Eligible patients were enrolled in a Medicare Advantage Prescription Drug plan for ≥ 12 months before and after the index date (first observed evidence of CKD). Patients with end-stage renal disease, kidney transplant, or hospice election preindex were excluded. Recommended comorbid disease management included hemoglobin A1c monitoring; adherence to glucose-lowering, cardiovascular, and angiotensin-converting enzyme inhibitors/angiotensin receptor blocker medications; and nephrologist/primary care provider (PCP) visits. HCRU was evaluated for 12 months postindex.

**Results:**

The final cohort of 241,628 patients was 55% female and 77% White, with an average age of 75 years. Approximately 90% of patients had Stage 3 CKD. Half had both diabetes and hypertension, and most of the remaining half had hypertension without diabetes. Patients meeting the criteria for good disease management, compared with patients not meeting those criteria, were less likely to experience an inpatient hospitalization, by as much as 40% depending on the criterion and the comorbidities present, or an emergency department visit, by as much as 30%. Total monthly healthcare costs were as much as 17% lower.

**Conclusions:**

Management of comorbid diabetes and hypertension in patients with CKD was associated with lower HCRU and costs. Care coordination programs targeting patients with CKD must give careful attention to glucose and blood pressure control.

**Trial registration:**

Not applicable.

## Background

Approximately 37 million people, representing 15% of U.S. adults, have chronic kidney disease (CKD) [1]. The two most common causes of CKD are diabetes and high blood pressure, and nearly 1 in 3 people with diabetes and 1 in 5 people with high blood pressure have kidney disease [2]. According to the Chronic Kidney Disease Surveillance System, only half of adults who are at high risk of kidney failure within 5 years (≥ 15% based on the Kidney Failure Risk Equation), defined as CKD Stage 3 or Stage 4 on the basis of laboratory measures of estimated glomerular filtration rate (eGFR), are aware they have kidney disease [3]. Despite the high prevalence of CKD, there is low awareness among individuals with CKD regarding their own risk for worsening disease. Further, there is a lack of understanding in the CKD population of how and why comorbid diabetes and hypertension contribute to development and progression of kidney disease [4, 5]. The confluence of these issues highlights the importance of closing gaps in care.

Current best-practice guidelines from the Kidney Disease: Improving Global Outcomes (KDIGO) organization include recommendations for managing both diabetes and hypertension [6, 7]. It is well-established that improved glycemic control in patients with diabetes has a beneficial effect on clinical markers for nephropathy [8]. The Diabetes Control and Complications Trial (DCCT) demonstrated that intensive glucose control in patients with type 1 diabetes resulted in a significant reduction in the risk of developing microalbuminuria and clinical albuminuria (macroalbuminuria), which are markers for nephropathy and risk factors for CKD [9, 10]. Similarly, for adults living with type 2 diabetes, the benefit of intensive glucose control on both the incidence and progression of microalbuminuria and macroalbuminuria has been demonstrated in various clinical trials [11, 12]. Further, for individuals with CKD and hypertension, blood pressure control is important for slowing CKD progression as well as reducing cardiovascular disease risk [13].

While the positive effects of managing comorbid disease on clinical CKD outcomes are well accepted, less is known regarding how these benefits translate to healthcare resource utilization (HCRU). In 2019, overall Medicare costs for people with CKD were estimated at $87.2 billion, or approximately $24,453 per Medicare beneficiary older than 65 years [14]. An improved understanding of how integrated disease management affects utilization is crucial for improving outcomes for individuals with CKD and preserving resources in the wider healthcare system. The objective of this study was to evaluate the association between effective management of comorbid diabetes and/or hypertension and HCRU and costs in patients with CKD.

## Methods

### Study design

This retrospective cohort study used the Humana Research Database to identify patients 19–89 years of age who were enrolled in a Medicare Advantage Prescription Drug (MAPD) plan and who had claims’ evidence of CKD Stage 3a or greater during the identification period (calendar year 2017). The Humana Research Database includes patients’ enrollment records, full medical and pharmacy claims, basic demographic information such as age, sex, and geographic region, detailed information on diagnosed medical conditions and procedures performed, and linked outpatient pharmacy records and lab results. Eligible patients had at least two outpatient medical claims with a diagnosis of CKD (ICD-10 diagnosis codes N18.3, N18.4 and N18.5), at least one inpatient medical claim with a diagnosis of CKD, or at least two serum creatinine laboratory values 90 days apart that translated to an eGFR value less than 60 ml/min/1.73m^2^. CKD stage was determined based on the eGFR value, and the diagnoses code in the absence of an eGFR. Eligible patients were enrolled in an MAPD plan for at least 12 months prior to the index date (for measuring baseline characteristics, including the CKD diagnosis) and at least 12 months following the index date (for measuring outcomes). The index date was the claims date providing the first evidence of CKD, and the observation period for receipt of guideline-recommended care was within 1 year of the index date. We operationalized the five indicators based on internal clinician input, study team experience and guidelines from the Practical Approach to Detection and Management of Chronic Kidney Disease for the Primary Care Clinician [15]. For HbA1c monitoring, the ADA guidelines [16] recommend that HbA1c should be measured at least twice a year when meeting treatment goals. Since we could not measure actual glycemic targets/HbA1c values for all patients in the study we used 2 measures of monitoring to consider someone as regularly following up and monitoring their glycemic targets. For adherence, typically a threshold of 80% proportion of days covered is considered adherent (i.e., 292/365 days). The study team selected no less than 60 days (or 2 months) per year which would translate to a PDC of 83%. For clinical care, we selected at least 3 nephrologist visits for high renal risk and at least 2 PCP visits among low renal risk based on the practical approach to management of CKD suggested by Vassalotti et al. [15].

**Fig. 1:**
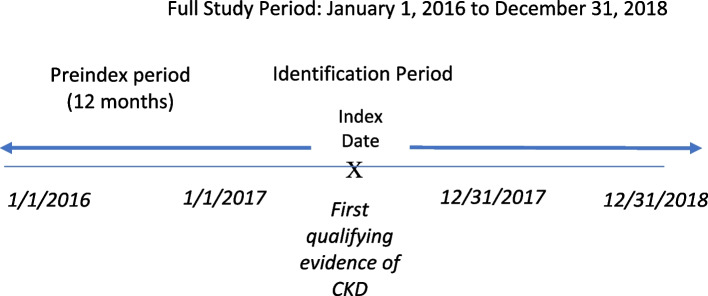
Study time frame

Patients with end-stage renal disease diagnosis codes on inpatient or outpatient medical claims or procedure codes indicating receipt of dialysis, recipients of kidney transplant, and patients who elected hospice care were excluded. Figure 1 displays the study time frame.

### Study outcomes

HCRU was measured by inpatient (IP) hospital admissions and emergency department (ED) visits during the 12 months postindex using facility claims. IP admissions were identified based on bill type code (11) and revenue codes (0190–0219). IP episodes were delineated by admit and discharge cates recorded on the claims. ED visits were identified using revenue codes (045x), place of service codes (23), and Current Procedural Terminology (CPT)/Healthcare Common Procedure Coding System (HCPCS) (CPT/HCPCS) codes indicating procedures performed in the ED setting. For both IP and ED, we created dichotomized outcomes indicating any use during the 12-month follow-up period. For cost outcomes, we calculated total medical costs by summing up allowed costs for all paid medical claims, and we calculated total pharmacy costs by summing up prescription costs for all paid pharmacy claims. Total healthcare costs were calculated by summing up medical and pharamacy costs.

### Explanatory variables

#### Baseline patient characteristics

CKD stages were based on eGFR values, which were either derived from serum creatinine laboratory results and defined according to the 2009 Chronic Kidney Disease Epidemiology Collaboration (CKD-EPI) equation [17], or were based on ICD-10 diagnosis codes N18.3, N18.4 and N18.5. Medical and pharmacy claims’ evidence was used to determine the presence of diabetes (type 1 or type 2) or hypertension. Other baseline characteristics included age, sex, race, geographic region, population density, Medicare Advantage plan type, eligibility for low-income subsidy or Medicaid coverage and Elixhauser Comorbidity Index (Table 1) [18–20]. The Elixhauser Comorbidity Index uses 31 categories of ICD-9 and ICD-10 diagnosis codes to calculate a score that is associated with hospital charges, length of stay, and mortality.

**Table 1:** Demographic and clinical characteristics of patients with chronic kidney disease

Characteristics	Total CKD Cohort
**N**	241,628
Age, mean (SD)	75.23 (± 7.97)
Female (n, %)	131,838 (54.6)
Geographic Region (n, %)	
Northeast	4480 (1.9)
Midwest	41,086 (17.0)
South	173,447 (71.8)
West	22,615 (9.4)
Population Density (n, %)	
Urban	161,821 (67.0)
Suburban	52,899 (21.9)
Rural	19,545 (8.1)
Unknown	7363 (3.0)
Race (n, %)	
White	184,816 (76.5)
Black	44,548 (18.4)
Other	11,086 (4.6)
Unknown	1178 (0.5)
Low Income Subsidy or Dual Eligibility (n, %)	65,474 (27.1)
Plan type (n, %)	
HMO	136,737 (56.6)
PPO/POS	78,945 (32.7)
FFS	8362 (3.5)
Other	17,584 (7.3)
Elixhauser Comorbidity Index, mean (SD)	5.22 (± 2.92)
Kidney Disease Stage (n, %)	
Stage 3a	162,555 (67.3)
Stage 3b	54,557 (22.6)
Stage 4	23,038 (9.5)
Stage 5	1,478 (0.6)

#### Indicators of good disease management

Primary independent variables were five indicators of good disease management that were assessed in the preindex period: 1) hemoglobin A1c (HbA1c) monitoring, identified by the presence of at least 2 HbA1c laboratory tests among those with evidence of diabetes; 2) adherence to glucose lowering medications, defined as fewer than 60 days off therapy, among those with evidence of diabetes; 3) adherence to cardiovascular therapy, defined as fewer than 60 days off therapy, among those with evidence of hypertension; 4) adherence to angiotensin-converting enzyme inhibitors/angiotensin receptor blockers (ACEi/ARBs), defined as fewer than 60 days off therapy, among those with evidence of proteinuria according to recorded urine albumin-to-creatinine ratio or a diagnosis code; and 5) clinical care, defined as at least 3 nephrologist visits among those at high renal risk or at least 2 primary care provider (PCP) visits among those at low renal risk. High renal risk was defined by eGFR < 30 and/or albuminuria ≥ 300 mg/g, as recommended in guidance for primary care physicians on when to refer CKD patients to a nephrologist [15].

### Statistical analysis

Baseline demographic and clinical characteristics were summarized for the study sample. Means and standard deviations were reported for continuous variables and counts and percentages for categorical variables. Regression analyses were conducted to examine the association between disease management and outcomes, while controlling for patient demographic characteristics, comorbidities, and CKD stage. Logistic models were used for HCRU outcomes, and generalized linear models with a gamma distribution and log link function were used for cost outcomes. All analyses were conducted separately for the four sub-cohorts: patients with both diabetes and hypertension, diabetes only, hypertension only, or neither. Statistical significance was assessed at the conventional level of 0.05.

## Results

**Fig. 2:**
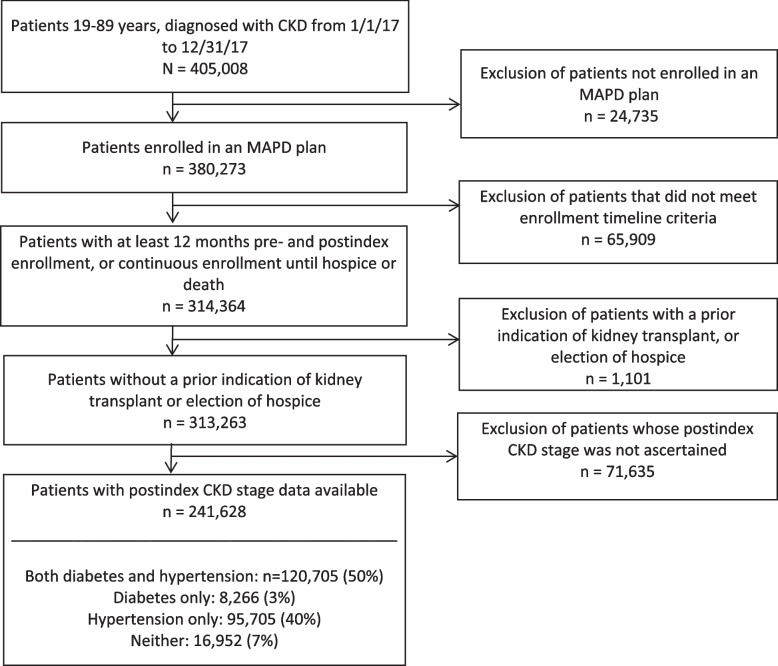
CKD cohort attrition

The final study cohort comprised 241,628 individuals (Fig. 2), half of whom had both diabetes and hypertension. Table 1presents patient characteristics. The group was 55% female and 77% White, with a mean age of 75 years. The majority of female patients in our population aligns with existing data, which demonstrate CKD is more common in women than men in the U.S [1, 21]. Approximately 27% of patients received a Low Income Subsidy for prescription drugs or were dually eligible for Medicare and Medicaid. Ninety percent of patients had Stage 3 kidney disease, meaning they had experienced mild to severe kidney damage. The remaining 10% of patients had Stage 4 to Stage 5 disease (kidneys close to failure or failed). Substantial comorbidity burden was indicated by a mean Elixhauser Comorbidity Index of 5.22.

Adjusted odds ratios (ORs) for the association between good disease management and HCRU outcomes are shown in Table 2. Among patients with diabetes or diabetes plus hypertension, the strongest associations were a 40% reduction in the odds of an IP admission and a 30% reduction in the odds of an ED visit when the HbA1c monitoring criterion was met. In these patients, adherence to glucose-lowering therapy was associated with a slightly greater than 10% reduction in both utilization outcomes. Patients with diabetes or diabetes plus hypertension also were less likely to have an IP admission or ED visit if they met the disease management criteria for either nephrologist or PCP visits, but most results for the provider visit indicators were not statistically significant in the group with diabetes only.

**Table 2:** Adjusted odds ratios [95% Confidence Intervals] for the impact of good comorbidity disease management on inpatient hospitalizations and emergency department visits in patients with chronic kidney disease

Indicators of Good Disease Management	Both Diabetes and Hypertension (*n* = 120,705)		Diabetes, No Hypertension (*n* = 8,266)		Hypertension, No Diabetes (*n* = 95,705)		Neither Diabetes Nor Hypertension (*n* = 16,952)	
	**Any IP Admission**	**Any ED visits**	**Any IP Admission**	**Any ED visits**	**Any IP Admission**	**Any ED visits**	**Any IP Admission**	**Any ED visits**
HbA1c monitoring, patients with diabetes	**.601 [.579-.624]**	**.702 [.676-.729]**	**.563 [.493-.644]**	**.670 [.591-.760]**	N/A	N/A	N/A	N/A
Glucose-lowering therapy adherence, patients with diabetes	**.897 [.873-.921]**	**.892 [.870-.914]**	**.886 [.799-.984]**	**.864 [.788-.946]**	N/A	N/A	N/A	N/A
CVD therapy adherence, patients with hypertension	**.930 [.901-.960]**	**.958 [.931-.986]**	N/A	N/A	**.901 [.872-.932]**	**.915 [.888—.943]**	N/A	N/A
ACEi/ARB adherence, patients with proteinuria	.986 [.927–1.049]	1.003 [.947–1.063]	1.060 [.745–1.506]	1.201 [.876–1.647]	1.002 [.893–1.123]	1.012 [.913–1.120]	.843 [.524–1.355]	.846 [.552–1.295]
≥ 3 nephrologist visits, patients at high renal risk	**.903 [.869-.938]**	**.934 [.901-.969]**	.832 [.412–1.682]	.771 [.393–1.514]	**.676 [.601-.761]**	**.791 [.710-.882]**	**.211 [.101-.440]**	**.400 [.227-.705]**
≥ 2 PCP visits, patients at low renal risk	**.735 [.676-.798]**	**.843 [.779-.912]**	**.791 [.687-.911]**	.975 [.857–1.110]	**.851 [.814-.889]**	**.886 [.851-.924]**	**.704 [.647-.767]**	**.835 [.774-.901]**

*ACEi/ARB* Angiotensin-converting enzyme inhibitors/angiotensin receptor blockers, *CVD* Cardiovascular disease, *ED* Emergency department, *HbA1C* Hemoglobin A1C, *IP* Inpatient admission, *N/A* Not applicable, *PCP* Primary care provider. Estimates adjusted for patient demographic characteristics, comorbidities, and CKD stage. Bolding denotes statistical significance at the 0.05 level

For patients with hypertension only, relevant disease management criteria were significantly associated with a reduction in both IP admissions and ED visits, except for the ACEi/ARB adherence criterion. Statistically significant reductions in the odds of utilization ranged from approximately 10% to 33%, depending on the disease management criterion and outcome measure. Among patients with neither diabetes nor hypertension, consistently significant reductions in utilization were associated with the two provider visit criteria. The other disease management criteria are specific to diabetes and hypertension, and therefore not relevant for this subgroup.

The association between good disease management and cost outcomes are shown in Table 3. In the subgroup of patients with both hypertension and diabetes, the patients who met HbA1c monitoring, glucose-lowering therapy, and provider visit criteria, had significantly lower total per person per month (PPPM) costs, relative to patients not meeting these criteria. The cost reductions ranged from 3% for glucose-lowering therapy to 17% for HbA1c monitoring. No association between meeting the cardiovascular therapy adherence or ACEi/ARB criteria and total PPPM costs was observed in this subgroup. In the diabetes only subgroup, significant associations with reduced total PPPM costs were detected for the HbA1c monitoring (26% reduction) and PCP visit (8% reduction) criteria. In the hypertension only subgroup, patients who met the cardiovascular therapy adherence and provider visit criteria had a 3% to 14% reduction in total PPPM costs, relative to patients not meeting these criteria. In the subgroup with neither diabetes nor hypertension, meeting the two provider visit criteria was associated with 19% to 45% reductions in total PPPM costs. This finding is in agreement with data suggesting proactive care of patients with CKD can reduce healthcare resource utilizations (i.e., hospitalizations and ED visits) [22] and lower costs [23].

**Table 3:** Mean ratio estimates [95% Confidence Intervals] for the impact of comorbidity disease management on total, medical, and pharmacy costs in patients with chronic kidney disease

Indicators of Good Disease Management	Both Diabetes and Hypertension (*n* = 120,705)	Diabetes, No Hypertension (*n* = 8,266)	Hypertension, No Diabetes (*n* = 95,705)	Neither Diabetes Nor Hypertension (*n* = 16,952)
**HbA1c monitoring, patients with diabetes**				
PPPM Total Costs	**.830 [.815 -.844]**	**.736 [.689-.786]**	N/A	N/A
PPPM Medical Costs	.**755 [.740 -.771]**	**.661 [.613-.713]**	N/A	N/A
PPPM Pharmacy Costs	**1.173 [1.149—1.197]**	.985 [.912–1.065]	N/A	N/A
**Glucose-lowering therapy adherence, patients with diabetes**				
PPPM Total Costs	**.969 [.958 -.980]**	.977 [.932–1.024]	N/A	N/A
PPPM Medical Costs	**.937 [.924 -.949]**	**.912 [.864-.963]**	N/A	N/A
PPPM Pharmacy Costs	**1.037 [1.022 -1.051]**	**1.078 [1.020–1.140]**	N/A	N/A
**CVD therapy adherence, patients with hypertension**				
PPPM Total Costs	1.007 [.993–1.021]	N/A	**.975 [.960-.991]**	N/A
PPPM Medical Costs	**.948 [.933-.963]**	N/A	**.954 [.938-.970]**	N/A
PPPM Pharmacy Costs	**1.222 [1.202 -1.242]**	N/A	**1.074 [1.053–1.096]**	N/A
**ACEi/ARB adherence, patients with proteinuria**				
Total Costs	.994 [.966–1.021]	1.172 [.996–1.379]	.998 [.945–1.054]	.900 [.705–1.148]
PPPM Medical Costs	.987 [.956–1.020]	1.184 [.983–1.425]	1.010 [.953–1.071]	.873 [.676–1.128]
PPPM Pharmacy Costs	1.011 [.978–1.045]	.994 [.820–1.205]	.959 [.896–1.026]	1.227 [.892–1.687]
** ≥ 3 nephrologist visits, patients at high renal risk**				
PPPM Total Costs	**.896 [.863-.930]**	1.216 [.855–1.729]	**.855 [.807-.907**	**.548 [.400 -.750]**
PPPM Medical Costs	**.843 [.807-.880]**	1.096 [.734–1.638]	**.804 [.755-.855]**	**.437 [.314—.608]**
PPPM Pharmacy Costs	**1.076 [1.029–1.126]**	**1.580 [1.043–2.393]**	**1.154 [1.074–1.240]**	.962 [.634 -1.460]
** ≥ 2 PCP visits, patients at low renal risk**				
PPPM Total Costs	**.954 [.938—.971]**	**.923 [.864—.987]**	**.917 [.897-.937]**	**.808 [.774-.843]**
PPPM Medical Costs	**.941 [.922—.960]**	.930 [.861—1.004]	**.904 [.883-.925]**	**.828 [.792-.866]**
PPPM Pharmacy Costs	1.016 [.995—1.037]	.939 [.869—1.015]	**.969 [.943-.996]**	**.727 [.687-.770]**

*ACEi/ARB* Angiotensin-converting enzyme inhibitors/angiotensin receptor blockers, *CVD* Cardiovascular disease, *HbA1C* Hemoglobin A1C, *N/A* Not applicable, *PPPM* Per person per month, *PCP* Primary care provider. Estimates were adjusted for patient demographic characteristics, comorbidities, and CKD stage. Bolding denotes statistical significance at the 0.05 level

For patients with both hypertension and diabetes, the patients who met HbA1c monitoring, glucose-lowering therapy, cardiovascular therapy, and nephrologist and PCP provider visit criteria had significantly lower medical PPPM costs, relative to patients not meeting these criteria. The medical cost reductions ranged from 5% for cardiovascular therapy to 24% for HbA1c monitoring. In the diabetes-only subgroup, significant associations with reduced medical PPPM costs were detected for HbA1c monitoring (34% reduction) and glucose-lowering therapy (9% reduction). In the hypertension-only subgroup, the patients who met cardiovascular therapy, nephrologist visit, and PCP visit criteria had significantly reduced medical PPPM. The cost reductions ranged from 5% for cardiovascular therapy to 20% for nephrology visit criteria. Across most groups, pharmacy PPPM costs increased.

## Discussion

CKD is common and frequently occurs with comorbid diabetes and hypertension. HbA1c monitoring, adherence to glucose-lowering and CVD medications, and regular physician visits are crucial to the management of CKD among these patients. This study found that meeting best practices of effective disease management in patients with CKD was generally associated with 1-year reductions in HCRU and in total and medical costs. Higher pharmacy costs were typically associated with good disease management. This observation is to be expected since good control of both glucose levels and blood pressure requires adherence to medications. The consistent relative cost reduction across total and medical PPPM categories is expected to more than offset increased pharmacy PPPM costs.

Findings from the present study complement prior research showing how the interplay between CKD and comorbid conditions affects utilization and costs. A recent study showed the occurrence of cardiovascular or renal hospitalization to be associated with greater all-cause HCRU and total costs [24] in patients with type 2 diabetes. In another study, higher 1-year rates of diabetes-related inpatient visits were associated with more versus less advanced CKD [25]. Higher total 1-year HCRU and medical costs have been observed in patients with CKD compared with patients without CKD [26]. Collectively, this body of research suggests managing comorbid clinical factors that can contribute to CKD progression, i.e., glucose levels and blood pressure, is important for improving patient outcomes and reducing the economic burden of CKD.

The reduction in HCRU and in overall costs observed in this study among patients with well-managed comorbidities may signal a reduction in CKD progression and/or a reduction in clinical conditions that would eventually contribute to CKD progression. The benefits of comorbid disease management could accumulate over time. Study findings not only help demonstrate the validity of current practice guidelines for comorbid disease management in patients with CKD [6, 7], they also suggest that careful attention to glucose and blood pressure control may yield measurable benefits for patients in the form of fewer IP stays and ED visits.

Effective management of patients with CKD who also have diabetes and/or hypertension requires a multifaceted approach that addresses screening, patient education, and comprehensive care. Interventions may target lifestyle change, medication adherence, regular follow-ups and monitoring of glucose level and blood pressure. Practical policies should also aim to reduce patient barriers to guideline-directed care and could include programs to solve for challenges such as transportation and language barriers.

### Limitations

Since health plan administrative databases are built primarily for billing and reimbursement purposes, clinical information extracted from these databases may be incomplete or inaccurate. Certain diagnostic information documented in medical records may not be fully captured in submitted claims. As a result, CKD cases, comorbid conditions, and/or outcomes could be undercounted or misclassified. Without access to medical records, it is difficult to gauge the extent of such occurrences. However, since we were comparing outcomes between patient groups and it is unlikely these billing errors or omissions would differ systematically between groups, the impact on our results should not result in systematic bias. Potential misclassification of patients into the low renal risk category due to limited lab data on albuminuria and glomerular filtration rate can occur. Additionally, disease management was operationally defined using process measures (e.g., HbA1c monitoring) rather than actual clinical outcomes (e.g., HbA1c control).​ We utilized the older CKD-EPI equation [17]. given the study period preceded 2021, however, future analysis of post-2021 data should utilize the 2021 CKD-EPI equation [27]. No causal inference can be ascertained from this study, as it was an observational design using retrospective claims data. The results may reflect healthcare patterns unique to Humana beneficiaries. This study utilized data for the Humana MAPD population only, so the results may not be generalizable to individuals with Traditional Medicare or to a general adult population. However, Humana is a large national health plan with members residing in a broad array of geographic regions.

## Conclusions

Management of diabetes and hypertension in patients with CKD was associated with lower HCRU and costs. This suggests a more coordinated and intentional focus on comorbid conditions for individuals living with CKD may yield measurable benefits to individuals, in the form of fewer hospitalizations and ED visits, and to the system in terms of reduced resource use and costs. Programs to educate patients with CKD on the benefits of managing diabetes and hypertension may support improved outcomes. Policies to support care coordination programs targeting patients with CKD must give careful attention to glucose and blood pressure control and should incentivize clinician adherence to guideline-directed CKD care as well. Future directions for research should investigate how the benefits of comorbid disease management could accumulate over time and which policies provide the most robust and reproducible reductions in HCRU and costs.

## Data Availability

The datasets generated and analyzed during the current study are not publicly available due to the proprietary nature of the work but may be available in summary form from the corresponding author on reasonable request.

## Abbreviations

ACEi: Angiotensin-converting enzyme inhibitors
ARB: Angiotensin receptor blockers
CKD: Chronic kidney disease
CKD-EPI: Chronic Kidney Disease Epidemiology Collaboration
CPT: Current Procedural Terminology
CVD: Cardiovascular disease
DCCT: Diabetes Control and Complications Trial
ED: Emergency department
eGFR: Estimated glomerular filtration rate.
HbA1c: Hemoglobin A1c
HCPCS: Healthcare Common Procedure Coding System
HCRU: Healthcare resource utilization
ICD: International Classification of Diseases
IP: Inpatient
KDIGO: Kidney Disease: Improving Global Outcomes
MAPD: Medicare Advantage Prescription Drug plan
PCP: Primary care provider
PPPM: Per person per month
